# Book Review

**Published:** 1901-09

**Authors:** 


**Manuali Hœpli.** Milano. 1901.—These manuals are individual factors in a series of 700 Manuali Hœpli, which collectively constitute a vast encyclopædia of science, letters and arts, the largest which has yet appeared in Italian. On a much smaller scale, the red-cover series in England, published by Messrs. Cassell and Co., are a similar group to the white-covered manuals published by the enterprising firm of Ulrico Hœpli, at Milan.

**Igiene della bocca e dei denti.** Del Dott. Ludovico Coulliaux. Pp. xiii., 300.—This little manual on the Hygiene of the mouth and teeth is very complete in itself. The mouth is taken in the different stages of dentition; the order of eruption, the various anomalies, and the more important points in anatomy and development are dealt in turn.

Without going into minute details of surgery, each disease is mentioned, and the principles of treatment indicated, and the rationale of the procedure explained.

The book is one which would well bear translating, and would be very useful for a student or general practitioner; and the subject is daily becoming of increasing importance, inasmuch as it is now believed that a neglect of the mouth is the beginning of many alimentary and blood diseases.

**L'assistenza dei pazzi nel manicomio e in famiglia.** Del Dott. A. Pieraccini, con prefazione del prof. E. Morselli. Pp. xvi., 262.—The care of the lunatic in asylums and at home. In the preface, Prof. Morselli points out the importance of regarding these cases as medical patients, and not merely as social prisoners; he expresses the opinion that, with proper treatment, the percentage of recoveries is increased. The first chapter defines very clearly and in simple language the different manifestations of lunacy, such as impulse, delusion, etc. The next few chapters detail the various departments of an asylum, and explain how they should be managed, with sections for melancholics, suicides, etc.

Rather more explicit is the article on the Infirmary. Finally, a chapter explains concisely how a patient can best be controlled in a private house.

The whole is very pleasant reading; it is quite suprising how much has been got into the space. For anyone, this manual should prove interesting and useful.

**Manuale di Chirurgia Operatoria.** Del Dott. R. STECCHI e A. GARDINI. Pp. viii., 322.—This is an excellent little manual. It does not treat of all the major operations of surgery, but of most of the more ordinary. It is well up to date and clear and concise. The anatomy of each operation is made a great point of, and the indications for the various operations are briefly indicated. The arrangement of the book is such as is usual in works on operative surgery. We rather take exception to Fig. 17, as certainly in England the director is no longer used by modern surgeons for the purpose indicated. We think the operation of Mikulicz might well have been omitted in a small book of this kind, and it seems a pity that no mention is made of ovariotomy or hysterectomy. A number of operations are described for the radical cure of femoral and inguinal hernia, and a very radical operation is recommended for removal of the breast for cancer. The surgery of the kidney is dealt with rather briefly. Excision is mentioned as a course to follow when possible in dealing with fistula in ano, and also as a modification of Wheelhouse's operation for stricture of the urethra when that operation is indicated. On the whole, so far as it goes, this book is a nice, compact little work, containing a great deal of information in a small space.

**Manuale del Massaggio.** Del Dott. ROMOLO MAJNONI. Pp. xii., 179.—After a brief description of the technique of massage, this manual gives the treatment of diseases of the joints and the muscular and nervous systems. To gynæcological massage is allotted a short chapter, illustrating the different kinds of manipulations in cases of chronic metritis, retroflectio and prolapsus uteri, and others. A somewhat more detailed description is given to the treatment, by means of vibrations, of certain affections of the mucous membrane of the nose, pharynx, and larynx. This mode of treatment, introduced, or at least made more generally known, by the Austrian physicians, Braun and Laker, is recommended in cases of different forms of rhinitis, in chronic pharyngitis and laryngitis. It is applied by means of sounds, and the vibrations are produced either by contraction of the arm-muscles of the operator, or a special vibratory apparatus. The end of the sound is generally wrapped in cotton-wool, soaked in some medicinal drug suitable for the case in question, so that the treatment cannot be said in every case to be purely mechanical. The book contains numerous illustrations.

